# HIV-Associated Cutaneous Kaposi's Sarcoma

**DOI:** 10.7759/cureus.13544

**Published:** 2021-02-24

**Authors:** Abdulrahman F Alluhaybi, Nael M Hatatah

**Affiliations:** 1 Dermatology, University of Hail College of Medicine, Hail, SAU; 2 Dermatology, King Salman Hospital, Riyadh, SAU

**Keywords:** kaposi's sarcoma, hiv, cutaneous

## Abstract

Kaposi's sarcoma (KS) is a vascular tumor that originates from the endothelial and immune cells. Lesions usually appear on the skin and oral mucosa, but they may also extend to involve lymph nodes and visceral organs. Patients typically present with multiple painless purplish spots on the face, oral mucosa, and genitalia.

We report a case of cutaneous KS in a 31-year-old male with an unknown positive HIV status. Clinical presentation and investigations were both toward KS. Therefore, our patient was treated immediately after diagnosis but could not tolerate the antiretroviral therapy and had unfortunate consequences.

## Introduction

Kaposi's sarcoma (KS) is a vasoformative spindle-cell tumor associated with human herpesvirus-8 (HHV-8, also known as Kaposi's sarcoma-associated herpesvirus [KSHV]) infection. There are four main clinical epidemiological types: epidemic-acquired immunodeficiency syndrome (AIDS)-related KS, classic sporadic KS, iatrogenic KS, and endemic KS [1]. The most common type is the epidemic KS, which affects HIV-positive patients. All variants of KS can occur at any age, but males are more commonly affected [2]. The overall incidence of KS in the general population was 1.53 per 100,000 persons among HIV-free populations, whereas the incidence of KS in HIV-positive individuals was found to be 481.54 per 100,000 persons [3]. In this report, we present a case of a 31-year-old male with multiple asymptomatic cutaneous violaceous plaques, which led to the diagnosis of HIV-related cutaneous KS.

## Case presentation

**Figure 1:**
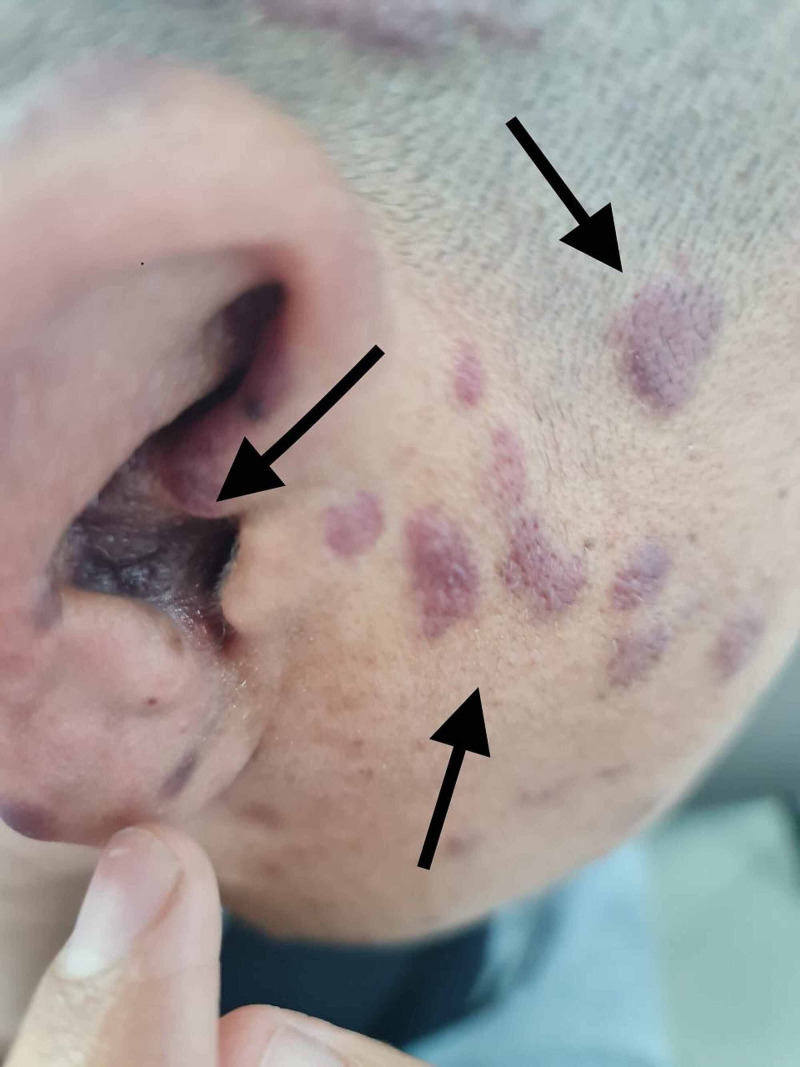
Multiple purple Kaposi's sarcoma plaques on the auricle and face

**Figure 2:**
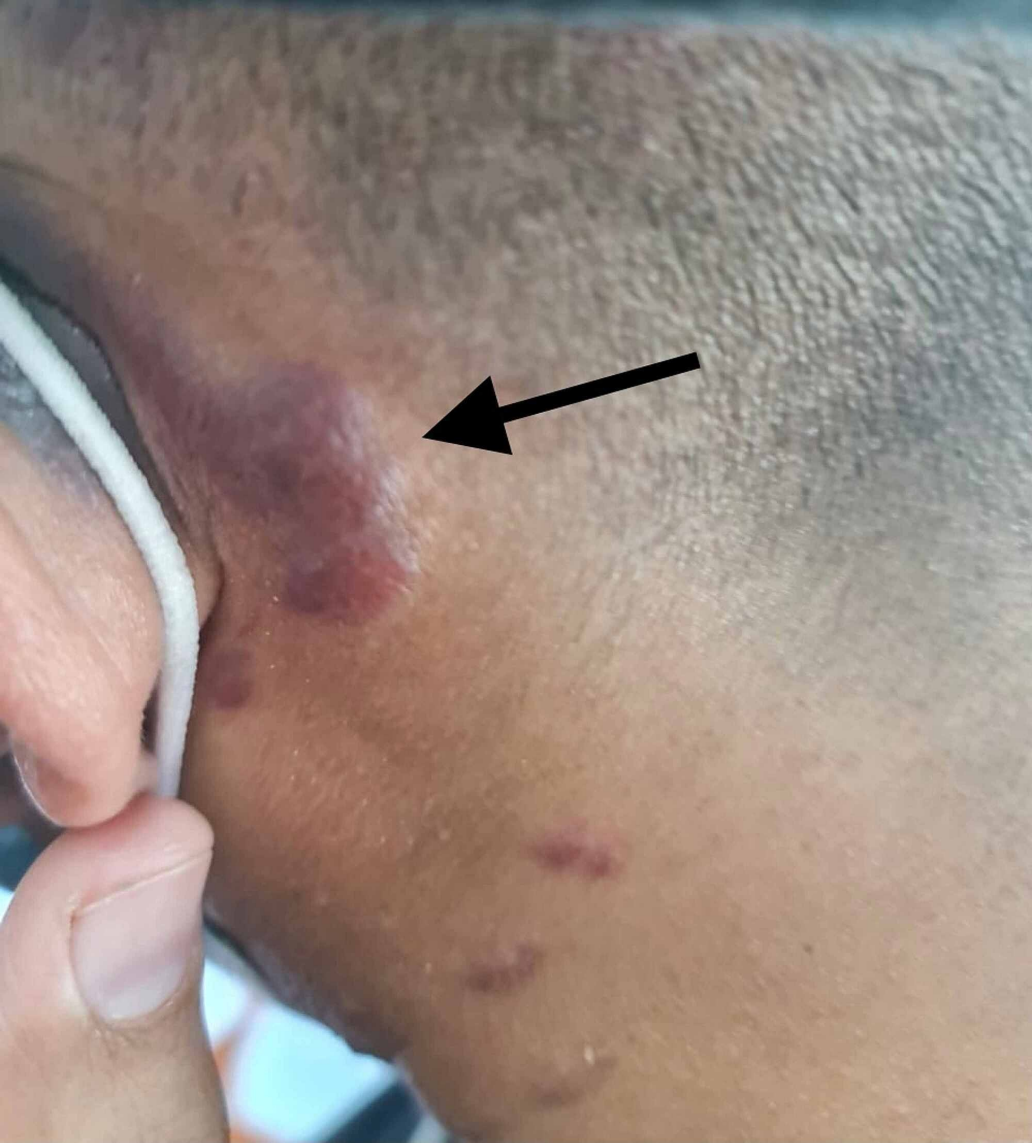
Erythematous to violaceous plaque posterior to the ear

**Figure 3:**
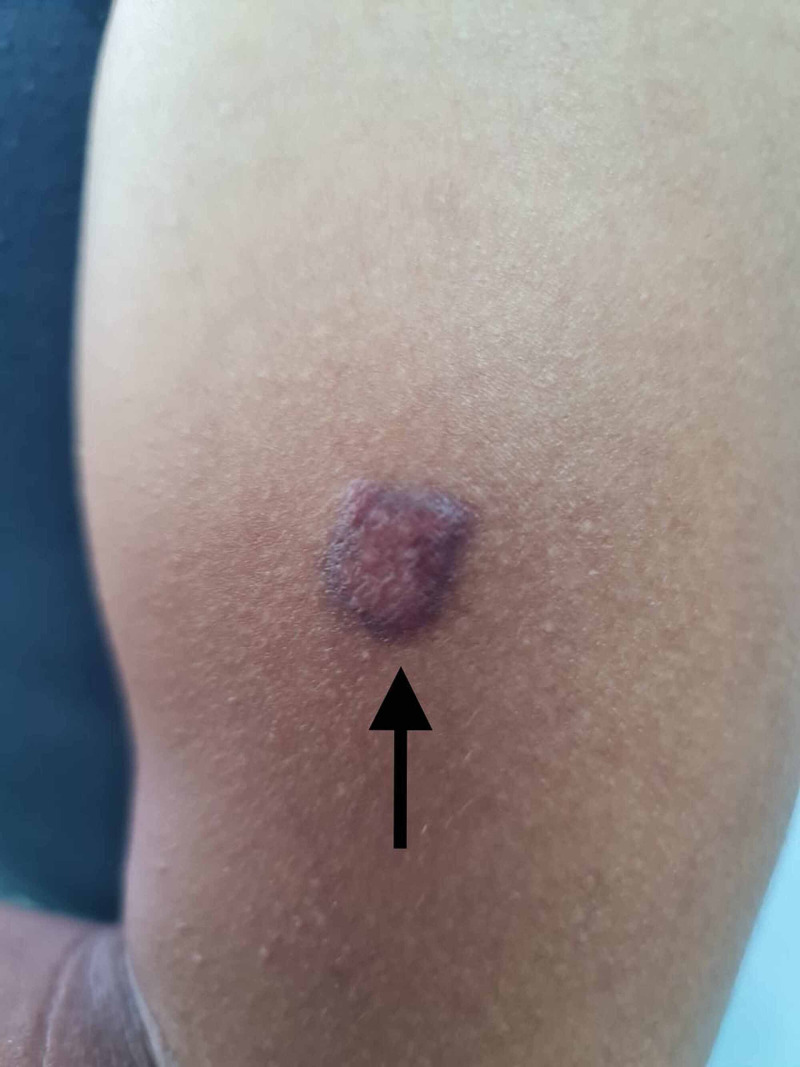
Purple-brown plaque on the upper arm

**Figure 4:**
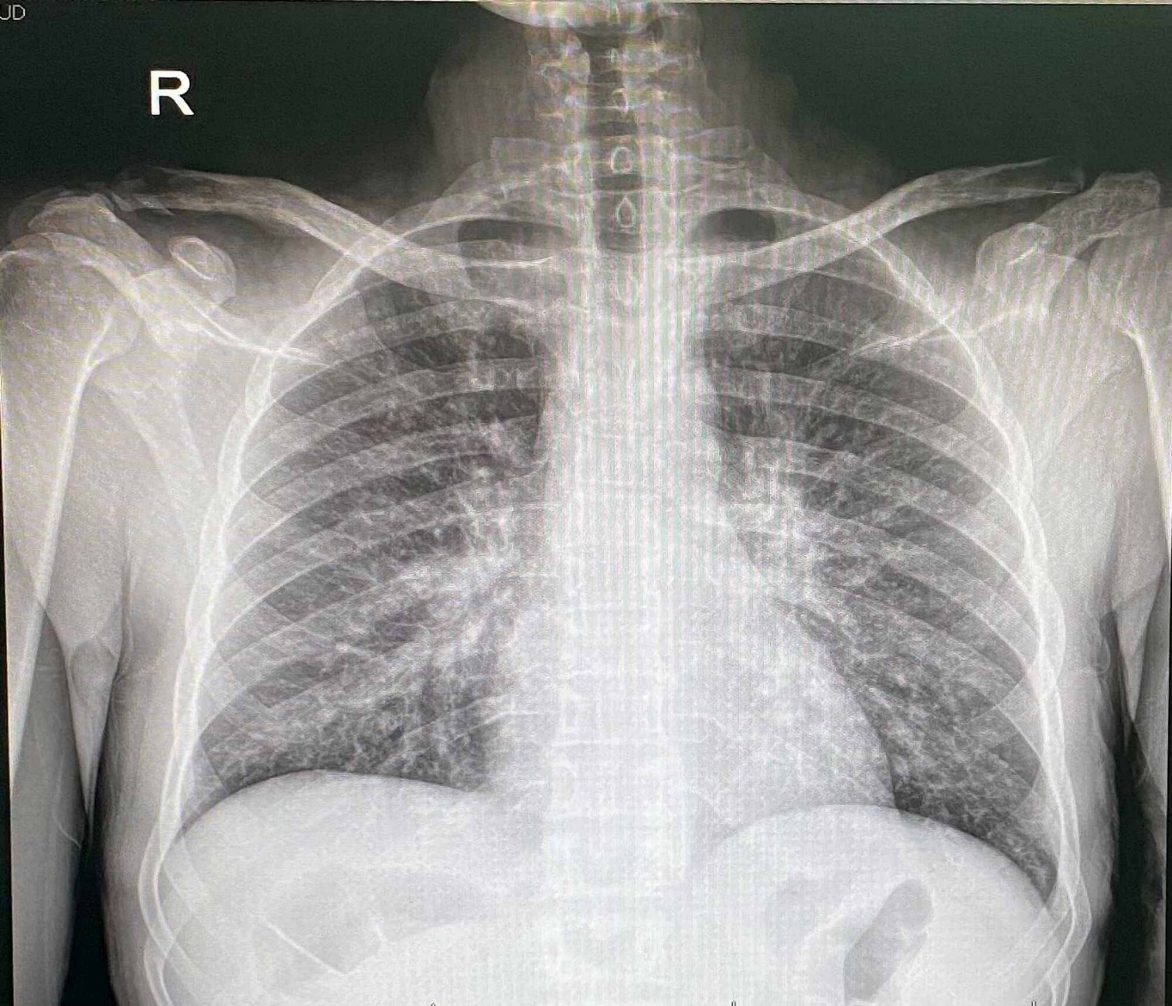
Posteroanterior chest radiograph showing bilateral reticulonodular infiltration

A 31-year-old male presented to the dermatology clinic with complaints of multiple non-pruritic lesions over the scalp, face, trunk, and arms that started six months ago and gradually disseminated. Otherwise, the patient’s past medical and drug history were unremarkable. On physical examination, there were multifocal violaceous nodules and plaques on the auricle, scalp, face, trunk, and arms (Figures 1-3). Lesions were varying in size and asymmetrically distributed. The patient had no other symptoms, and there was no history of weight loss and gastrointestinal tract bleeding. Lymphadenopathy and lymphedema were also not noticed. A skin biopsy measuring 4 x 3 x 3 mm was taken for histopathological microscopic examination. The histopathologist reported a diffuse dermal infiltrative vasoformative lesion extending to the base of the biopsy, with extravasated red blood cells noted in the background. There was no granulomatous inflammation, and features of lupus erythematosus were also not seen. HHV-8 latency-associated nuclear antigen (LANA-1) within the spindle cells was also detected by immunohistochemical staining. A series of hematological Investigations were carried out. Blood tests were normal except having a decrease in both absolute neutrophils and CD4+ counts. Kidney and liver function tests were also normal. Moreover, HIV test was also performed showing a positive HIV result. Hence, clinical presentation, laboratory workup, and histopathological report were all consistent with KS. Soon after the diagnosis was confirmed, the patient was initiated on an antiretroviral combination regimen (elvitegravir, cobicistat, emtricitabine, and tenofovir) and topical immunomodulator. Three weeks after commencing the patient on an antiviral regimen, the patient developed immune reconstitution inflammatory syndrome (IRIS). He could not pursue his usual daily activities due to severe muscle pain and cough. Chest X-ray was ordered and revealed bilateral reticulonodular opacities more in the left lower zone, which indicated pulmonary infection that supported the diagnosis of IRIS (Figure 4). Consequently, the patient was advised to stop his antiretroviral regimen and was referred to a tertiary hospital with better facilities for further investigations and better therapeutic options.

## Discussion

KS was firstly described in 1872 as an idiopathic, multi-pigmented skin lesion [4]. There is a significant link between KS development, HHV-8 infection, and HIV infection. The 10-year probability of developing KS was approximately 50% among HIV-infected patients who were infected with both HIV and HHV-8 [5]. HIV-related KS is more common in men having sex with men (MSM), injection drug users, and transfusion recipients [6]. There are 10 morphological variants of KS lesion: patch, plaque, nodular, lymphadenopathic, exophytic, infiltrative, ecchymotic, telangiectatic, keloidal, and cavernous or lymphangioma-like. The skin lesion ranges from several millimeters to centimeters in diameter and is able to grow rapidly within a few weeks [7].

Although KS can be sometimes diagnosed clinically by the appearance of lesion, biopsy should be taken to confirm the diagnosis. KS has been observed in almost all visceral sites, and therefore any patient with KS should be evaluated for any visceral involvement [8]. Treatment is similar for all KS types, but it should be individualized according to the severity and widespread of lesion. Supportive care for nutritional and psychosocial well-being is required for these patients for better quality of life. There are multiple local treatment options that can be used for cosmesis and the management of limited KS lesions. Small lesions can be regressed by intralesional chemotherapy. Vinblastine is the most frequently used agent [9]. Furthermore, a topical gel (alitretinoin) can be applied to treat cutaneous KS lesions, but it is rarely used due to its considerable drug interactions and side effects [10].

Irrespective of CD4 cell count, initiation of antiretroviral therapy (ART) is highly recommended for all HIV-infected patients. ART is the cornerstone in the treatment of HIV-related KS [11]. A marked decrease in both the incidence and severity of newly diagnosed KS in HIV-infected patients coincided with the increased use of ART [2,12]. However, reactivation of indolent infections was demonstrated following the initiation of ART in a condition known as IRIS [13].

## Conclusions

In HIV patients not receiving ART, KS is a progressive disease with a considerable mortality rate. Nevertheless, diagnostic delay can lead to more opportunistic infections, which increase the risk of developing IRIS. Early cutaneous KS lesions can be easily mistaken as hematomas, purpura, angiomas, or nevi. Hence, our case emphasizes the importance of considering HIV-associated cutaneous KS as a differential diagnosis of any multiple painless violaceous skin lesions.
